# Oral antibiotics for neonatal infections: a systematic review and meta-analysis

**DOI:** 10.1093/jac/dkz252

**Published:** 2019-06-24

**Authors:** Fleur M Keij, René F Kornelisse, Nico G Hartwig, Irwin K M Reiss, Karel Allegaert, Gerdien A Tramper-Stranders

**Affiliations:** 1 Department of Pediatrics, Division of Neonatology, Erasmus MC-Sophia Children’s Hospital, Rotterdam, The Netherlands; 2 Department of Pediatrics, Franciscus Gasthuis & Vlietland, Rotterdam, The Netherlands; 3 Department of Development and Regeneration, KU Leuven, Leuven, Belgium

## Abstract

**Background:**

Worldwide many neonates suffer from bacterial infections. Adequate treatment is important but is associated with prolonged hospitalization for intravenous administration. In older children, oral switch therapy has been proven effective and safe for several indications and is now standard care.

**Objectives:**

To evaluate the currently available evidence on pharmacokinetics, safety and efficacy of oral antibiotics and oral switch therapy in neonates (0–28 days old).

**Methods:**

We performed systematic searches in Medline, Embase.com, Cochrane, Google Scholar and Web of Science. Studies were eligible if they described the use of oral antibiotics in neonates (0–28 days old), including antibiotic switch studies and pharmacological studies.

**Results:**

Thirty-one studies met the inclusion criteria. Compared with parenteral administration, oral antibiotics generally reach their maximum concentration later and have a lower bioavailability, but in the majority of cases adequate serum levels for bacterial killing are reached. Furthermore, studies on efficacy of oral antibiotics showed equal relapse rates (OR 0.95; 95% CI 0.79–1.16; *I*^2^ 0%) or mortality (OR 1.11; 95% CI 0.72–1.72; *I*^2^ 0%). Moreover, a reduction in hospital stay was observed.

**Conclusions:**

Oral antibiotics administered to neonates are absorbed and result in adequate serum levels, judged by MICs of relevant pathogens, over time. Efficacy studies are promising but robust evidence is lacking, most importantly because in many cases clinical efficacy and safety are not properly addressed. Early oral antibiotic switch therapy in neonates could be beneficial for both families and healthcare systems. There is a need for additional well-designed trials in different settings.

## Introduction

Infections remain a main cause of morbidity and mortality among newborns.^1^ Early-onset sepsis, defined as a proven bacterial infection in the first 72 h of life, has an overall incidence of ∼1/1000 live births, with a higher incidence in premature and/or very-low-birthweight infants.^2^ Forty-five percent of all childhood mortality under 5 years occurs in the neonatal period, of which 22% is due to neonatal infections, including pneumonia.^3^

Early diagnosis remains challenging due to non-specificity of both clinical symptoms and laboratory findings.^4^ When bacterial infection is probable or proven, parenteral antibiotics are usually prescribed for at least 7 days.^5^ Occasionally, when intravenous (iv) access problems occur, or when hospital referral is not possible, as in low-and-middle-income countries (LMICs), newborns are treated with oral antibiotics. In high-income countries (HICs), the full course is generally completed iv.

Intravenous therapy and thus prolonged hospitalization interferes with parent–child bonding and is associated with other hospital-related risks and substantial costs.^6^^,^^7^ In older children, oral switch therapy, defined as a switch to oral antibiotics within a treatment course once the patient is clinically well, has been proven to be effective and safe for a variety of indications and is now part of standard practice.^8^

The adequacy of antibiotic treatment depends on its specific pharmacological mode of action. Efficacy of penicillins and cephalosporins, both commonly used drugs in neonatology, depends on *T*_>__MIC_. For vancomycin, efficacy depends on AUC/MIC and for aminoglycosides it depends on *C*_max_. The MIC is pathogen specific and cut-off values vary by antibiotic.^9^^,^^10^

To our knowledge, no systematic review evaluating the use of oral antibiotics in neonates has been performed. Together with the uncertainties regarding oral absorption in the first weeks of life, the lack of evidence may be a possible reason why oral switch therapy is not yet standard care in neonates. The aim of this systematic review is therefore to evaluate the currently available evidence on safety and efficacy of iv-to-oral switch therapy in neonates, and to evaluate whether, following oral antibiotic administration, adequate serum concentrations are attainable in neonates (0–28 days).

## Methods

### Search strategy and study selection

We performed a systematic review in accordance with the Preferred Reported Items for Systematic Reviews and Meta-analysis (PRISMA),^11^ searching Medline, Embase.com, Cochrane Central, Google Scholar and Web of Science on 22 February 2019. The PRISMA statement and full search strategies can be found in the Supplementary data (available at *JAC* Online). Titles and abstracts were screened and the full text of potential articles was reviewed independently by two reviewers (F. M. K. and G. A. T.-S.). Disagreements were resolved by discussion or through consultation with a third investigator (R. F. K.). Congress abstracts, reference lists and reviews were screened for additional studies. Eligible studies were limited to those performed in humans. Since we expected the amount of evidence to be small, we did not apply any restriction regarding year of publication or language. We included randomized controlled trials (RCTs), intervention studies and retrospective studies describing the use of oral antibiotics including oral switch therapy and pharmacological studies in newborns 0–28 days of age.

The protocol was registered in PROSPERO (protocol number CRD42017070854).

### Data extraction

Three authors (F. M. K., G. A. T.-S. and K. A.) independently extracted the data following a predefined extraction form (see Supplementary data). We did not contact authors for additional information.

### Quality assessment

Quality assessment was performed independently by two authors (F. M. K. and either K. A. or G. A. T.-S.) using the Cochrane Risk of Bias Tool for RCTs^12^ and the Newcastle–Ottawa Quality Assessment Scale (NOS) for non-randomized trials.^13^ Since a tool for quality assessment of pharmacological papers is currently lacking, we used the ClinPK statement, a descriptive tool without a grading system, to assess quality of pharmacokinetics papers (Table S2).^14^

### Data analysis

When possible, data were pooled to assess efficacy of oral treatment. We calculated pooled ORs with 95% CI using Review Manager V5.3. Heterogeneity was assessed using *Q* statistics and *I*^2^ values and interpreted following the thresholds of the Cochrane Handbook for Systematic Reviews of Interventions.^15^^,^^16^ A fixed-effects model was applied when heterogeneity was low (*I*^2^ <40%), otherwise a random-effects model was used. We performed a sensitivity analysis based on indication for antibiotic treatment. In addition, a subgroup analysis was performed with respect to the clinical indication and antibiotic regimen.

## Results

**Figure 1.**
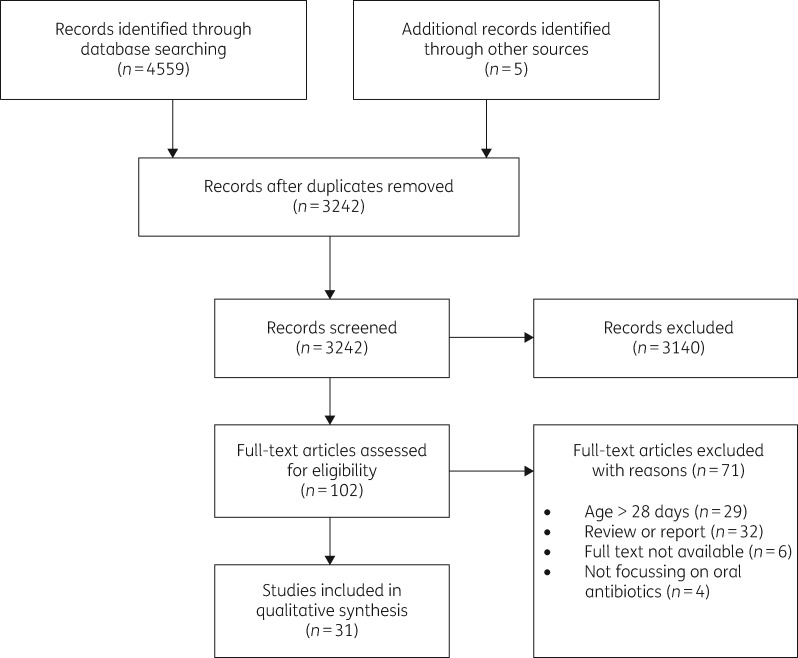
Study selection.

From a total of 4559 studies, we reviewed the full text of 102 potential articles. Figure 1 shows the selection process. Additionally, five articles were selected through screening of reference lists, leading to 31 selected publications for this review. The characteristics of included studies are described in Table 1.

**Table 1:** Characteristics of included studies

Author	Country	Study design	Study size	Participant and infection characteristics	Intervention group	Type of antibiotic	Comparison group	Primary aim	Primary outcome
***Assessment of pharmacokinetics***									
*Healthy subjects*									
Huang and High (1953)^17^	USA	non-RCT	unknown	healthy (pre)term newborns	single dose of oral antibiotics	penicillin	single dose of im antibiotics	comparison of absorption rate	(i) mean serum levels
O’Connor *et al.* (1965)^18^	USA	cohort study	*n* = 15	healthy newborns (PNA 0–2 days)	oral antibiotics	nafcillin	no comparison	serum levels following oral therapy	(i) mean serum levels
Grossman and Ticknor (1965)^19^	USA	non-RCT	*n* = 171	healthy term newborns (PNA 0–5 days)	single dose of oral antibiotics	nafcillin, cloxacillin, ampicillin	single dose of im antibiotics	comparison of serum levels following oral/im	(i) mean serum levels
Weingärtner *et al.* (1977)^20^	Germany	cohort study	*n* = 23	healthy preterm/term newborns	single dose of oral antibiotics	amoxicillin	no comparison	serum level determination	(i) mean serum levels
*Neonates with clinical indication for antibiotic therapy*									
Silverio and Poole (1973)^21^	USA	case–control	*n* = 10	term newborns (GA 40 weeks; PNA 1–2 days), clinical indication	single dose of oral antibiotics	ampicillin	oral antibiotics in adults	comparison of serum concentrations	(i) mean serum levels
Cohen *et al.* (1975)^22^	Scotland	non-RCT	*n* = 27	newborns (GA 28–40 weeks, PNA 1–6 days), prophylactics/UTI	oral antibiotics	ampicillin, amoxicillin, flucloxacillin	no comparison	determination of serum concentrations of oral antibiotics	(i) mean serum levels
Lönnerholm (1982)^23^	Sweden	crossover trial	*n* = 14	newborns, suspected infection, good clinical condition	iv-to-oral switch	amoxicillin, ampicillin	no comparison	determination of bioavailability of oral antibiotics	(i) mean serum levels
Mulhall (1985)^24^	England	non-RCT	*n* = 9	newborns (GA 34.6 ± 2 weeks, PNA 14 ± 3 days), sepsis	oral antibiotics	chloramphenicol	iv antibiotics	comparison of oral/iv antibiotic therapy	(i) mean steady-state concentration
Herngren *et al.* (1987)^25^	Sweden	cohort study	*n* = 9	newborns (GA 36.6 weeks; PNA 7.2 days), suspected sepsis	iv-to-oral switch	flucloxacillin	no comparison	determination of kinetics of flucloxacillin	(i) pharmacokinetics of oral and iv antibiotics
									(ii) side effects
Weber *et al.* (1999)^26^	Philippines, The Gambia	non-RCT	*n* = 58 (*n* = 34: PNA <29 days)	newborns <3 months, severe bacterial infection	oral antibiotics	chloramphenicol	im	pharmacokinetics of chloramphenicol	(i) mean serum levels
***Assessment of pharmacokinetics and clinical efficacy***									
Squinazi *et al.* (1983)^27^	France	cohort study	*n* = 20	preterm/term newborns, suspected sepsis, 1–8 days PNA	oral antibiotics	amoxicillin	–	efficacy and tolerance of oral therapy	(i) clinical course
									(ii) tolerance
									(iii) serum levels
Autret *et al.* (1988)^28^	France	RCT	*n* = 21	full-term newborns (PNA 3** **days), bacterial colonization	oral antibiotics	amoxicillin	iv amoxicillin	comparison of serum levels iv/oral with MIC	(i) serum levels >MIC
									(ii) clinical course and tolerance
Autret (1989)^29^	France	cohort study	*n* = 10	full-term newborns (GA 39.8 ± 1.8 weeks) bacterial colonization, clinically well	iv-to-oral antibiotic switch after 48 h	amoxicillin	no comparison	*C*_max_ and steady-state concentrations in relation to MIC cut-off values	(i) serum levels >MIC
									(ii) accumulation
									(iii) clinical course and tolerance
Giustardi and Coppola (1992)^30^	Italy	RCT	*n* = 32	term newborns (GA 39–40 weeks, PNA 2–3 days), neonatal sepsis	oral antibiotics	amoxicillin	iv amoxicillin	comparison of serum levels	(i) mean serum levels
									(ii) clinical course
Gras le Guen *et al.* (2007)^31^	France	cohort study	*n* = 222	term newborns (GA 39.2±1.5 weeks; PNA 2 days), possible or proven early-onset GBS sepsis	iv-to-oral antibiotic switch after 48 h iv therapy	amoxicillin	no comparison	reaching adequate serum levels and tolerance of iv/oral switch therapy	(i) re-infection rate within 3 months
									(ii) tolerance
									(iii) serum levels
Mir (2013)^32^	Pakistan	pilot study of larger RCT	*n* = 44 (*n* = 29: PNA 0–27 days)	newborns (GA 38 weeks), clinical signs of severe infection	oral antibiotics	amoxicillin	no comparison	pharmacokinetic efficacy targets (*T*_>MIC_)	(i) dose–exposure profile, time–exposure profile *T*_>MIC_ ≥50%; MIC 2.0 mg/L
Sicard *et al.* (2015)^33^	France	retrospective study	*n* = 16	preterm newborns (GA: 28±3.5 weeks; PNA: 20.9±11.7 days) with a bacterial infection	oral antibiotics	linezolid	parenteral antibiotics	description of linezolid concentrations, clinical course and side effects in premature infants	(i) disappearance of clinical symptoms
									(ii) side effects
									(iii) plasma concentrations
***Assessment of clinical efficacy***									
Tikmani *et al.* (2017)^34^	Pakistan	RCT	*n* = 970 (*n* = 754: 0–28 days)	term newborns (GA>37 weeks, PNA 15.4 ± 16.2 days), fast breathing	oral antibiotics	amoxicillin	placebo	equivalence of oral amoxicillin compared with placebo	(i) treatment failure by day 8 post-enrolment visit
Mir *et al.* (2017)^35^	Pakistan	RCT	*n* = 2780 (*n* = 1083: 0–6 years)	newborns, clinical signs of severe infection	comparison of three regimens	(i) gentamicin + oral amoxicillin	procaine benzylpenicillin + gentamicin	assessment of equivalence of two regimens	(i) treatment failure within 7 days after enrolment
						(ii) procaine benzylpenicillin → oral amoxicillin			
Degefie Haielgebriel *et al.* (2017)^36^	Ethiopia	RCT	*n* = 22 geographical clusters, *n* = 11 intervention, *n* = 11 control	newborns with possible signs of serious infection	regimen of im + oral antibiotics	gentamicin im + oral amoxicillin	–	feasibility and mortality impact of a simplified antibiotic regimen	(i) post-day 1 neonatal mortality
Baqui *et al.* (2015)^37^	Bangladesh	RCT	*n* = 2490 (*n* = 253: 0–6 days)	newborns, clinical signs of severe infection	comparison of three regimens	(i) gentamicin im + oral amoxicillin.	procaine benzylpenicillin + gentamicin	identification of effective alternative antibiotic regimens	(i) treatment failure within 7 days after enrolment
						(ii) procaine benzylpenicillin + gentamicin im → oral amoxicillin			
Tshefu *et al.* (2015)^38^	DR Congo, Kenya, Nigeria	RCT	*n* = 2333 (*n* = 882: 0–6 days)	newborns, fast breathing	oral antibiotics	amoxicillin	injectable penicillin + gentamicin	effectiveness of oral amoxicillin compared with injectable procaine benzylpenicillin/gentamicin	(i) treatment failure by day 8 post-enrolment visit
Tshefu *et al.* (2015)^39^	DR Congo, Kenya, Nigeria	RCT	*n* = 3564 (*n* = 1160: 0–6 days)	newborns, clinical signs of bacterial infection	comparison of four regimens	(i) gentamicin + oral amoxicillin	procaine benzylpenicillin + gentamicin	effectiveness of simplified antibiotic regimens compared to injectable procaine benzylpenicillin/gentamicin	(i) treatment failure by day 8 post-enrolment visit
						(ii) procaine benzylpenicillin + gentamicin → oral amoxicillin			
						(iii) gentamicin + oral amoxicillin			
Zaidi *et al.* (2012)^40^	Pakistan	RCT	*n* = 434 (*n* = 333: 0–28 days)	newborn, possible serious bacterial infection	comparison of three regimens	(i) ceftriaxone im	procaine benzylpenicillin + gentamicin	comparison of failure rates of three clinic-based antibiotic regimens	(i) treatment failure within 7 days after enrolment
						(ii) oral co-trimoxazole			
Manzoni *et al.* (2009)^41^	Italy	case–control study	*n* = 108 (36/72)	full-term newborns, presumed/proven bacterial infection	iv-to-oral antibiotic switch	cefpodoxime	matched controls, continuation of iv therapy	efficacy, safety, tolerability of switch therapy	(i) clinical course (timing of normalization of laboratory data, duration of hospitalization, type of feeding)
Bang *et al.* (2005)^42^	India	case–control from previous study	*n* = 39 intervention villages, *n* = 47 control villages	newborns, clinical signs of possible infection	regimen of im + oral antibiotics	gentamicin im + oral co-trimoxazole	–	evaluation of feasibility and effectiveness of home-based management of neonatal sepsis	(i) neonatal sepsis related mortality
Bang *et al.* (1999)^43^	India	case–control study	*n* = 39 intervention villages, *n* = 47 control villages	newborns, clinical signs of possible infection.	regimen of im + oral antibiotics	gentamicin im + oral co-trimoxazole	–	reduction of neonatal mortality by introduction of neonatal home packages including antibiotics	(i) neonatal mortality rate
Blond *et al.* (1990)^44^	France	non-RCT	*n* = 119	term newborns + 6 preterm, bacterial colonization	iv-to-oral antibiotic switch after 3 days	amoxicillin, amoxicillin/clavulanic acid	–	efficacy of oral treatment	(i) clinical course in first month of life
Coffey *et al.* (2012)^45^	Nepal	cohort study	*n* = 67	newborns with possible severe bacterial infection	regimen of im + oral antibiotics	gentamicin im + oral co-trimoxazole	–	feasibility of gentamicin prefilled injection system + oral antibiotics	(i) clinical course(ii) local reaction to injection
Qamar *et al.* (2013)^46^	Pakistan	descriptive study	*n* = 1083	newborns, omphalitis	oral antibiotics	cefalexin	injectable procaine penicillin + gentamicin, topical gentian violet	description of clinical profile and outcome of home-based management	(i) decreased area of redness/cellulitis or purulent discharge
									(ii) complete resolution of signs of sepsis
									(iii) development of signs of sepsis
Magín *et al.* (2007)^47^	Spain	retrospective study	*n* = 172	newborns (PNA 7–31 days), UTI	iv-to-oral switch	amoxicillin/clavulanic acid	no comparison	examination of clinical course, efficacy of short-term iv therapy	(i) re-infection within 14 days after cessation of therapy

### Quality assessment

Risk of bias in seven out of nine RCTs was low; in the remaining two it was unclear (Figure S1).^28^^,^^30^ In all studies, blinding of patients and personnel was considered unethical [e.g. repeated intramuscular (im) placebo administration] and therefore not performed. However, the independent outcome assessors were blinded for treatment allocation. Seven RCTs were registered in a public trial register.^34–40^ The quality of the six observational papers was acceptable (Table S4). With regard to the pharmacological studies, with focus on pharmacokinetics, overall, quality seems adequate taking into account available methods of analysis at that time. However, in some cases crucial information was missing, such as gestational age (GA) or postnatal age (PNA), or the exact methods used (Table S3). The complete assessment is included in Table S1.

### Study population

As expected, the study population was quite heterogeneous, including both term and preterm infants of different postnatal ages. Four studies were performed in healthy newborns, admitted for a non-infectious indication.^17–20^ The remaining 27 studies included subjects with a clinical condition requiring antibiotics, ranging from prophylactic use to culture-proven infection. Two studies evaluated oral switch therapy in neonates with culture-proven sepsis.^31^^,^^41^ Thirteen studies were performed in LMICs. In these trials, antibiotic therapy indication was defined solely on clinical symptoms.^26^^,^^32^^,^^34–40^^,^^42^^,^^43^^,^^45^^,^^46^

### Absorption of oral antibiotics

#### Pharmacokinetic analysis and interpretation

In 10 papers serum levels were determined using the agar plate diffusion method; the remaining and more recently published papers used HPLC. Most studies provided descriptive data on absorption, mainly *C*_max_ without further pharmacokinetic estimates (e.g. *V* and CL). Three papers provided AUC estimates.^21^^,^^23^^,^^28^ Regarding interpretation, six papers reported MIC cut-off values^28–33^ with only one study reporting a *T*_>MIC_.^32^ Extracted pharmacokinetic data and administered doses are described in Table 2.

**Table 2:** Pharmacokinetic data on oral antibiotics

Study	Population	Type of antibiotics	Route (mode of administration)	Dose^a^	Timing between birth/admission and first oral antibiotic dose	Sampling schedule (h)	Mean *C*_max_	Mean *T*_max_ (h)	AUC (mg·h/L)
Huang and High (1953)^17^	(i) term newborns	procaine penicillin potassium penicillin G	oral vs im	22000 U/kg sd	–	½, 2, 4, 6	2.5 U/mL (1.0–4.0)	2	–
		potassium penicillin G					3.50 U/mL (0.5–8.0)	0.5	–
	(ii) premature infants	procaine penicillin potassium penicillin G					3.25 U/mL (0.5–16.0)	2	–
		potassium penicillin G					2.18 U/mL (0.5–4.0)	2	–
O’Connor *et al.* (1965)^18^	(i) newborns	nafcillin	oral (liquid preparation)	10 mg/kg sd	within 48 h	1, 2, 4, 6, 8, 12	2.559 mg/L	2	–
	(ii) newborns			15 mg/kg sd			5.491 mg/L	2	–
	(iii) children			12.5 mg/kg sd			4.076 mg/L	1	–
Grossman and Ticknor (1966)^19^	term newborns, healthy, <5 days old	nafcillin	oral (suspension)	10 mg/kg	<5 days	½, 1, 2, 3, 4, 6, 8, 10, 12, 24, 48 (max 6/patient)	7.2 mg/L	2	–
		cloxacillin					24.4 mg/L	1–2	–
		ampicillin					10.2 mg/L	3–4	–
Silverio and Poole (1973)^21^	(i) full-term infants	ampicillin	oral (drops)	10 mg/kg q6h	24–48 h	before, 2, 6, 12 h after	4.3 mg/L	6	36.8
	(ii) adults						3.2 mg/L	1.8	11.7
Lönnerholm *et al.* (1982)^23^	newborns, suspected/proven bacterial infection	pivampicillin	oral	50 mg/kg q12h	5–7 days	½, 2, 4, 8, 12	20.1 ± 2.0 mg/L	2	95 ± 10
		amoxicillin					27.3 ± 5.9 mg/L	2	145 ± 25
Herngren *et al.* (1987)^25^	newborns (33–41 weeks), suspected bacterial infection	flucloxacillin	oral (suspension)	50 mg/kg q12h	–	1 h before, 5 times in 12 h	69.8 ± 30 mg/L	–	–
Cohen *et al.* (1975)^22^	newborns, UTI/prophylactic antibiotics	ampicillin	oral (syrup)	25 mg/kg sd	<7 days	½, 2, 4, 6, 9, 12, 15, 18, 24, 36 (then daily)	6.9 ± 10.9 mg/L	9	–
		ampicillin/flucloxacillin		25 mg/kg sd			5.2 ± 5.6 mg/L	15	–
		flucloxacillin		25 mg/kg sd			15.8 ± 23.1 mg/L	2	–
		amoxicillin		30 mg/kg sd			5.2 ± 3.0 mg/L	4–9	–
Weingärter *et al.* (1977)^20^	(i) term newborns	amoxicillin	oral	50 mg/kg q6h	first days of life	2, 4, 6, 10, 24	38 mg/L ± 19	4	–
	(ii) premature infants						59 mg/L ± 13	4	–
Squinazi *et al.* (1983)^27^	term newborns, suspected bacterial infection	amoxicillin	oral (suspension)	75 mg/kg q12h	<3 days (*N* = 1 after 8 days)	1½, 3, 8, 12	32.7 ± 30.3 mg/L (3.3–118.3 mg/L)	3	–
Autret *et al.* (1988)^28^	term newborns, bacterial colonization	amoxicillin	oral	40 mg/kg q12h	iv-oral switch after 48 h	½, 2, 6, 9	31 ± 13.5 mg/L	2–6	305 ± 211 (163–924)
			iv				80.7 ± 32 mg/L	0	400 ± 298 (149–1145)
Autret (1989)^29^	term newborns (39.8 ± 1.8 weeks), bacterial colonization	amoxicillin	oral	25 mg/kg q6h	iv-oral switch after 48 h	2 h after first dose, 2 and 6 h after last dose	first dose: 22.2 ± 8.3 mg/L; last dose 2h 25.2 ± 7.6 mg/L; last dose 6 h 14.4 ± 7.6 mg/L	–	–
Giustardi and Coppola (1992)^30^	term newborns, suspected bacterial infection	amoxicillin	oral vs iv	40 mg/kg q12h	<1 day	½, 2, 6, 9	oral: 29.30 ± 12.75 mg/L	2	–
							iv: 68.59 ± 34.8 mg/L	0.5	–
Gras le Guen *et al.* (2006)^31^	newborns (>36 weeks) probable/proven GBS infection	amoxicillin	oral	300 mg/kg/day q6h	after 48 h	48	35.04 ± 18.93 mg/L (steady-state)	–	–
				200 mg/kg/day q6h			29.46 ± 17.74 mg/L (steady-state)	–	–
Mir (2013)^32^	infants 0–2 months with signs of sepsis (*n* = 29) 0–27 days	amoxicillin	oral	75–100 mg/kg/day q12h	directly	before, 23 h and 6–8 h after	2–3 h after: 11.6 ± 9.5 mg/L	–	–
							6–8 h after: 16.4 ± 9.3 mg/L	–	
Sicard *et al.* (2015)^33^	premature neonates, infection, switch to linezolid because of renal failure after vancomycin	linezolid	oral vs iv	10 mg/kg q8h	20.9 ± 11.7 days	7 ± 1.5 h after last dose	9.04 mg/L (0.69-32.9 mg/L)	–	–
Mulhall (1985)^24^	newborns with clinical sepsis	chloramphenicol	oral	43 ± 8 mg/kg/day q12h	–	1 h before, 2–3 h after	13.3 ± 4.2 mg/L	–	–
Weber *et al.* (1999)^26^	infants <3 months, possible severe infection (*n* = 19) <28 days	chloramphenicol	oral (*n* = 18) vs im (*n* = 16)	25 mg/kg <7 days sd, 7–29 days: q12h	directly	½, 1, 2, 3	½ of oral treated patients reached therapeutic range (10–25 mg/L)	–	–

a sd, single dose.

#### Penicillin

Penicillin, a narrow-spectrum β-lactam antibiotic, was the first oral antibiotic studied in neonates.^17^ A weight-equivalent dose was administered orally or im to small groups of healthy subjects of different age (preterm and term newborns, infants or children). This resulted in a lower *C*_max_ following oral compared with im administration in all age groups. Moreover, a higher AUC following oral administration was reported in newborns compared with older children.

#### Ampicillin/amoxicillin

Absorption of oral ampicillin and amoxicillin, both broad-spectrum β-lactam antibiotics, was evaluated in several studies in newborns (GA 28–40 weeks; PNA 0–6 days).^19–22^ Following im injection *T*_max_ was 30 min, whereas this was on average 4 h for oral therapy. Compared with adults, *C*_max_ was higher and was reached later in neonates, with even higher levels found in preterm newborns. A small switch study evaluated the bioavailability of ampicillin and amoxicillin, reporting lower plasma concentrations following oral administration compared with equivalent im doses (AUC oral/im, ampicillin 59%, range 22%–94%; amoxicillin 75%).^23^ A randomized study in neonates suspected of a bacterial infection compared oral with iv amoxicillin. Initial serum levels were higher in the iv group but comparable concentrations were reached 2 h after oral administration.^30^ Most recently a population pharmacokinetic study has been performed among 44 neonates receiving parenteral gentamicin combined with oral amoxicillin.^32^ Sampling 2–3 and 6–8 h after administration showed concentrations exceeding the susceptibility breakpoint for amoxicillin against *Streptococcus pneumoniae* (MIC 2.0 mg/L) strains at both timepoints, meaning that *T*_>MIC_ is >50% for a 12 h dosing interval.

#### Flucloxacillin/nafcillin

Levels of flucloxacillin and nafcillin, both narrow-spectrum β-lactam antibiotics, have been reported following single-dose administration and combined with other antibiotics to newborns (28–42 weeks GA; 0–6 days PNA). Both drugs appear to be absorbed faster than other penicillins, with a *T*_max_ of 2 h for both following oral administration.^18^^,^^19^^,^^22^ The corrected bioavailability of oral flucloxacillin (corrected for a change in terminal half-life) was reported to be 47.7%, which is almost equivalent to that in adults.^25^

#### Chloramphenicol

Chloramphenicol, a broad-spectrum antibiotic, is not generally used in neonatal care due to substantial side effects (e.g. grey baby syndrome).^48^ Plasma levels following identical oral and iv dose administration have been evaluated, showing a lower steady-state concentration following oral treatment (oral 13.3 mg/L; iv 25.7 mg/L).^24^ Similar results were found in a multicentre study, with only half of term infants reaching therapeutic levels (recommended range in study 10–25 mg/L) following oral administration (25–50 mg/kg/day q12h or q24h depending on PNA).^26^

### Efficacy of oral antibiotics

#### Amoxicillin

Amoxicillin is the most studied oral antibiotic in neonates with a probable or proven bacterial infection. Its efficacy depends on the *T*_>MIC_. In preterm and term newborns (PNA 1–8 days) with a probable bacterial infection, no relapse was reported after oral treatment (80–150 mg/kg/day q12h). Moreover, no side effects occurred and all measured serum concentrations were reported to be above the MICs of targeted pathogens.^27^^,^^30^ In a clinical study on *Escherichia coli* urinary tract infection (UTI), four neonates showed no re-infections in the next 2 years following a 14 day oral treatment of 120 mg/kg/day (in an era with low *E. coli* amoxicillin resistance).^22^ In an RCT including 21 neonates with suspected infection, 11 switched to oral amoxicillin (120 mg/kg q8h) after 48 h of iv therapy (ampicillin/netilmicin). The control group switched to amoxicillin iv. All patients included in the study had negative blood cultures and tolerated oral feeding well without any vomiting. Concentrations remained above the MIC for *E. coli* for all but three patients (*n* = 2 iv, *n* = 1 oral).^28^ Dose optimization through increasing the dosing frequency was suggested and subsequently evaluated in a second study. Ten infants switched to oral amoxicillin (100 mg/kg/day q6h). All plasma concentrations were above the MIC for *E. coli* without substantial side effects or re-infections.^29^

An uncontrolled iv-to-oral switch trial was performed in 222 term neonates with probable or proven group B-streptococcal (GBS) sepsis. Subjects switched to oral amoxicillin (300 mg/kg/day q6h) after 48 h of iv amoxicillin (100 mg/kg per day). All infants had to be asymptomatic and enterally fed at the moment of switch. Because of high serum concentrations, the dose was reduced (to 200 mg/kg/day q6h) in the remaining 158 patients. Serum levels were all above the MIC for GBS. Moreover, therapy was well tolerated without any side effects or reinfections and a reduction of 5 days in hospital admission was seen.^31^

#### Amoxicillin/clavulanic acid

A retrospective study evaluated the clinical course and treatment of 172 newborns with a UTI. An increase in use of oral instead of iv therapy was seen over the years. In total, 119 patients switched to oral amoxicillin/clavulanic acid (dose not reported) as continuation therapy. None of the orally treated newborns experienced a relapse in the 6 months after treatment.^47^ In another study, oral amoxicillin/clavulanic acid (80 mg/kg/day q12h) was administered successfully to neonates at risk of infection without any re-infections or treatment failure in the first month after treatment completion.^44^

#### Cefalexin

A study from Pakistan described the outcome of oral management in neonates with clinical omphalitis. Omphalitis was categorized based on severity; cases without sepsis were treated with cefalexin suspension (50 mg/kg/day q8h) with a success rate of 99.5%, showing that outpatient treatment of clinically well neonates with omphalitis using oral therapy is feasible.^46^

#### Cefpodoxime

Switching therapy from iv to oral was performed in 36 term neonates with a probable or proven bacterial infection. After 72 h of iv treatment (ampicillin/sulbactam + amikacin), patients who were asymptomatic switched to oral cefpodoxime (10 mg/kg/day), a third-generation cephalosporin. Seventy-two matched controls continued on iv therapy. Outcomes were comparable for the two groups, with identical inflammatory parameters in the first week of treatment and no mortality after 1 month. Admission duration was significantly lower and breastfeeding rate was significantly higher among neonates with an oral switch.^41^

#### Flucloxacillin

In a small switch study, performed in 1987, neonates at risk of sepsis switched to oral flucloxacillin combined with oral amoxicillin after severe bacterial infection had been ruled out. Plasma concentrations following oral administration were all above the MIC cut-offs for *Staphylococcus aureus*.^25^

#### Linezolid

In a retrospective study, five preterm infants (GA 28 ± 3.5 weeks), treated for late-onset sepsis, who experienced renal failure, switched from iv vancomycin (30 mg/kg/day) to oral linezolid (30 mg/kg/day q8h). *C*_max_ for all patients but one was above the measured MIC for the causative pathogen.^33^

### Larger efficacy studies including trials in LMIC settings

Since there is a need for good outpatient-based management in LMICs, several large trials have taken place evaluating regimens including oral antibiotics. In a controlled trial in >80 villages in India, health workers in the intervention villages were trained in providing neonatal care.^42^^,^^43^ When clinical sepsis was suspected but admission refused, neonates received home-based treatment including oral co-trimoxazole. Sepsis-related mortality decreased from 16.6% to 6.9% compared with the period before introduction. Subsequently, several large RCTs comparing home-based antibiotic regimens have been published. The evaluated regimens are described in Table 3.

**Table 3.** LMIC trials and antibiotic regimens

Author	Intervention	Control
Bang *et al*.^42^^,^^43^	gentamicin im + co-trimoxazole syrup	no treatment
Zaidi^40^	(i) ceftriaxone (50 mg/kg/day) im (7 days)	benzylpenicillin im + gentamicin im (7 days)
	(ii) oral co-trimoxazole (5 mg/kg q8h) + gentamicin im (7 days)	
Baqui *et al*.*^37^	(i) oral amoxicillin (50 mg/kg q12h) + gentamicin im (7 days)	benzylpenicillin im + gentamicin im (7 days)*
	(ii) benzylpenicillin + gentamicin im (2 days) followed by oral amoxicillin (5 days)*	
Tshefu *et al*.*^39^	(i) oral amoxicillin (50 mg/kg q12h) + gentamicin im (7 days)	benzylpenicillin im + gentamicin im (7 days)*
	(ii) benzylpenicillin + gentamicin im (2 days) followed by oral amoxicillin (5 days)	
	(iii) gentamicin im + oral amoxicillin (2 days) followed by oral amoxicillin (50 mg/kg q12h) (5 days)*	
Tshefu *et al*.^38^	oral amoxicillin (50 mg/kg q12h)	benzylpenicillin im + gentamicin im (7 days)
Mir *et al*.*^35^	(i) gentamicin im + oral amoxicillin (50 mg/kg q12h) (7 days)	benzylpenicillin im + gentamicin im (7 days)*
	(ii) procaine benzylpenicillin im + gentamicin (2 days) followed by oral amoxicillin (5 days)*	
Degefie Hailegebriel *et al*.^36^	oral amoxicillin (40 mg/kg q8h) + gentamicin im (7 days)	no treatment
Tikmani *et al*.^34^	oral amoxicillin (50 mg/kg q12h) (7 days)	placebo

* Included in the meta-analysis.

Three regimens were compared in 434 Pakistani children 0–59 days old (72% were ≤28 days old). Higher treatment failure rates were seen among patients treated with oral co-trimoxazole plus gentamicin compared with other regimens.^40^ In a Nepalese study, oral co-trimoxazole was administered in combination with im gentamicin to 67 newborns with a possible bacterial infection.^45^ The authors reported a 100% completion rate of oral therapy without any treatment failure. An Ethiopian trial evaluated the implementation of im gentamicin and oral amoxicillin.^36^ When infection was suspected, pre-referral medication was given and the patient was referred to the hospital. If referral was not possible, the intervention group continued with home-based treatment; the control group did not receive further treatment. Results seem promising, with a decline in mortality from 17.9 deaths per 1000 live births at baseline to 9.4 per 1000 in the intervention group. In the comparison group, mortality rates declined to a lesser extent, from 14.4 to 11.2 per 1000. However, mortality rates were not significantly lower in the intervention group compared with the control (*P* = 0.33).

**Figure 2.**
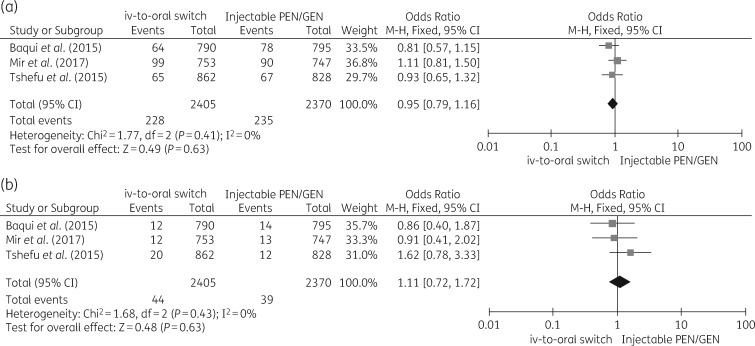
(a) Forest plot comparing treatment failure of reference treatment (penicillin/gentamicin im for 7 days) with switch regimen (penicillin/gentamicin im for 2 days followed by oral amoxicillin for 5 days). The regimens used are further described in Table 3. (b) Forest plot comparing mortality of reference treatment (penicillin/gentamicin im for 7 days) with switch regimen (penicillin/gentamicin im for 2 days followed by oral amoxicillin for 5 days). The regimens used are further described in Table 3. PEN, penicillin; GEN, gentamicin.

Three RCTs, with a total of 8834 subjects, compared regimens including oral amoxicillin with standard im regimens (penicillin/gentamicin) in newborns at risk of severe infection. The first trial, in Bangladesh, compared three regimens, including an oral switch regimen, among 2490 children (10% aged 0–6 days)^37^ The second trial, in the Democratic Republic of the Congo, Kenya and Nigeria (AFRINEST study) included 3564 infants 0–59 days old (30% 0–6 days old)^39^ comparing four regimens including one oral switch to amoxicillin. The third study included 2453 infants (44% 0–6 days of age) evaluating similar regimens.^35^ Heterogeneity between studies was low. Primary outcome was treatment failure within 8 days, defined as death, clinical deterioration, hospital admission or treatment-related serious adverse events. The combined OR for the orally treated group was 0.95 (95% CI 0.79–1.16; *I*^2^ 0%) Mortality within 2 weeks after enrolment was comparable in both groups, with an OR of 1.11 (95% CI 0.72–1.72; *I*^2^ 0%). Forest plots are shown in Figure 2.

Finally, two trials evaluated the use of oral amoxicillin in neonates with tachypnoea as a single symptom of possible infection. The first, in which oral treatment was compared with placebo in 849 infants (78% 0–28 days old; dropout: *n* = 121), showed a higher mortality in the placebo group compared with the treatment group, underlining the potential benefits of antibiotic treatment in infants with fast breathing alone.^34^ A second trial, including 2333 neonates (38% 0–6 days old), showed equivalence of oral amoxicillin compared with an im regimen in newborns with fast breathing, with comparable treatment failure rates [22% (im regimen) versus 19% (oral regimen)] and mortality rates (<1% in both groups).^38^

## Discussion

In this systematic review, we collected the currently available evidence on oral antibiotics in neonates. While oral administration is not commonly considered at present in neonates, several pharmacological and efficacy studies have been performed with different types of antibiotics.

In general, adequate serum levels according to the MICs of relevant pathogens can be achieved after oral administration in neonates. Inter-individual variation is observed, which has also been reported following iv administration and should therefore not be used as an argument for discarding oral therapy. *C*_max_ is reached later after oral administration compared with other routes. Thus, as in older patients, initial therapy should consist of iv antibiotics to quickly reach target concentrations, but can subsequently be switched to oral therapy once the neonate is clinically well.

The efficacy studies showed equal relapse rates and good toleration of oral therapy compared with iv therapy without reporting an increase in side effects. Moreover, in two studies oral administration led to a shorter stay in hospital and more exclusively breast-fed infants. In LMICs, mortality rates have decreased through the introduction of home-based therapy when referral is not possible and simplified antibiotic regimens with an oral switch have shown efficacy similar to that of standard im therapy.

The strength of this review is the fact that we provide a complete overview of all retrieved studies on oral antibiotic use in neonates. Although this provides a great historical overview of an idea that has existed since the 1950s, the heterogeneity of the studies found makes pooling and generalizability to current clinical practice difficult. In an attempt to translate findings to contemporary practice, limitations will be discussed in the light of study design and setting, ethics, techniques used and analysis.

First, study groups were small and without randomization, except for a few large RCTs, introducing a possible selection bias with exclusion of the sicker newborns. In most studies, clinical efficacy, bacterial re-infection or treatment failure is used as the primary outcome. Given the fact that the bacterial re-infection rate is low, a much larger study sample is needed to show non-inferiority or efficacy of oral treatment.^49^ Moreover, the clinical indication for antibiotic treatment and infection severity is unclear in a number of studies; therefore data cannot be translated to current practice.

The included studies were performed in both preterm and term infants, sometimes without providing the GA or PNA of the subjects. Drug clearance differs between preterm and term infants and improves with increasing postnatal age, thereby influencing plasma concentrations.^50^ Finally it must be stressed that a great variety of antibiotic regimens have been used, including single-dose administration, and sometimes without mentioning the administered dose. Some of the therapies and regimens are rarely prescribed nowadays, partly due to increased concerns regarding antibiotic resistance and the availability of alternatives with fewer side effects.

In LMICs, simplified regimens including oral antibiotics are already recommended by the WHO when referral is not possible.^51^ Unfortunately, the setting differs greatly from HICs, with refusal of hospital admission still being common and accepted, especially in remote areas. In addition to the differences in setting, the majority of patients are solely diagnosed on clinical symptoms since diagnostic tools are often lacking, possibly leading to an overestimation of the actual number of bacterial infections. Furthermore, the intensity of surveillance due to the execution of the study combined with exclusion of the sicker neonates may have biased mortality rate numbers.

Regarding the pharmacokinetic analysis, ethics requirements of studies have changed and the same holds true for the administration of antibiotics to healthy newborns. With regard to blood sampling, it is no longer considered ethical to collect large volumes or many samples in neonates. Advanced population pharmacokinetic approaches should be applied in further research, using a reduced number of samples per newborn.^52^

Further, improved knowledge and better techniques have led to novel antibiotic assays, replacing agar plate dilution methods. Advanced analysis programs are available in order to develop pharmacokinetic models, used for prediction of exposure and drug response, following different dosage regimens in a target population. Those models take into account covariates such as gestational and postnatal age or disease characteristics that possibly influence the pharmacokinetics and dynamics of a drug. Notably, none of the included papers reported covariates in their analysis.

Finally, for the interpretation of results and thus the evaluation of efficacy, the pharmacological mode of action of the specific antibiotic should be considered. The effect of β-lactam antibiotics depends on *T*_>MIC_, whereas for aminoglycosides it depends on the *C*_max_/MIC ratio. Although six papers do refer to MIC, only one reports *T*_>MIC_. Comparison of *C*_max_ with a single MIC value in case of β-lactam antibiotics has no clinical relevance and cannot be used as a relevant surrogate marker for therapy efficacy. Moreover, MIC levels have increased in recent years, due to an increase in bacterial resistance. In 1992, Giustardi and Coppola^30^ reported an amoxicillin MIC of 5 mg/L for *E. coli*, whereas now an MIC of >8 mg/L is advised to properly treat an *E. coli* infection. Given these limitations, the currently published studies cannot be used as conclusive evidence to safely change our current guidelines on management of neonatal bacterial infection. However, our findings do give the impression that such studies may be undertaken safely.

### Conclusion and future directions

Early switch to oral antibiotics after a short course of iv antibiotics could be promising in term neonates with a (probable) bacterial infection. This claim is partly supported by the available evidence retrieved in this systematic review. Unfortunately, the lack of large well-designed studies in a high-income setting, evaluating the efficacy of oral antibiotics, together with the uncertainties regarding pharmacokinetics has obstructed further implementation. Future research should focus on the clinical efficacy of oral therapy and the safety of iv-to-oral antibiotic switch therapy in neonates using different types of antibiotics, taking into account the mode of action of the specific antibiotic. These studies should include pharmacokinetic analyses when possible, to properly evaluate currently used dosing regimens. Once iv-to-oral switch therapy is proven to be safe and effective in neonates, its implementation may have a strong effect on health-cost reduction and quality of life.

## Supplementary Material

dkz252_Supplementary_Data
