# Supplementary material for: Oral antibiotics for neonatal infections: a systematic review and meta-analysis
Source: J Antimicrob Chemother. 2019 Jun 24;74(11):3150–61. doi: 10.1093/jac/dkz252 (PMC6814091; doi:10.1093/jac/dkz252)

**Supplementary data**

**PRISMA statement**

| **Section/topic** | **#** | **Checklist item** | **Reported on page #** |
| --- | --- | --- | --- |
| **TITLE** | | |  |
| Title | 1 | Identify the report as a systematic review, meta-analysis, or both. | 1 |
| **ABSTRACT** | | |  |
| Structured summary | 2 | Provide a structured summary including, as applicable: background; objectives; data sources; study eligibility criteria, participants, and interventions; study appraisal and synthesis methods; results; limitations; conclusions and implications of key findings; systematic review registration number. | 2 |
| **INTRODUCTION** | | |  |
| Rationale | 3 | Describe the rationale for the review in the context of what is already known. | 3 |
| Objectives | 4 | Provide an explicit statement of questions being addressed with reference to participants, interventions, comparisons, outcomes, and study design (PICOS). | 3 |
| **METHODS** | | |  |
| Protocol and registration | 5 | Indicate if a review protocol exists, if and where it can be accessed (e.g., Web address), and, if available, provide registration information including registration number. | 4 |
| Eligibility criteria | 6 | Specify study characteristics (e.g., PICOS, length of follow-up) and report characteristics (e.g., years considered, language, publication status) used as criteria for eligibility, giving rationale. | 4 |
| Information sources | 7 | Describe all information sources (e.g., databases with dates of coverage, contact with study authors to identify additional studies) in the search and date last searched. | 4 |
| Search | 8 | Present full electronic search strategy for at least one database, including any limits used, such that it could be repeated. | Suppl material |
| Study selection | 9 | State the process for selecting studies (i.e., screening, eligibility, included in systematic review, and, if applicable, included in the meta-analysis). | 4-5 |
| Data collection process | 10 | Describe method of data extraction from reports (e.g., piloted forms, independently, in duplicate) and any processes for obtaining and confirming data from investigators. | 4 |
| Data items | 11 | List and define all variables for which data were sought (e.g., PICOS, funding sources) and any assumptions and simplifications made. | 4 |
| Risk of bias in individual studies | 12 | Describe methods used for assessing risk of bias of individual studies (including specification of whether this was done at the study or outcome level), and how this information is to be used in any data synthesis. | 4 |
| Summary measures | 13 | State the principal summary measures (e.g., risk ratio, difference in means). | 4-5 |
| Synthesis of results | 14 | Describe the methods of handling data and combining results of studies, if done, including measures of consistency (e.g., I^2^) for each meta-analysis. | 4-5 |

Page 1 of 2

| **Section/topic** | **#** | **Checklist item** | **Reported on page #** |
| --- | --- | --- | --- |
| Risk of bias across studies | 15 | Specify any assessment of risk of bias that may affect the cumulative evidence (e.g., publication bias, selective reporting within studies). | N.A. |
| Additional analyses | 16 | Describe methods of additional analyses (e.g., sensitivity or subgroup analyses, meta-regression), if done, indicating which were pre-specified. | N.A. |
| **RESULTS** | | |  |
| Study selection | 17 | Give numbers of studies screened, assessed for eligibility, and included in the review, with reasons for exclusions at each stage, ideally with a flow diagram. | 6 & figure 1 flow chart |
| Study characteristics | 18 | For each study, present characteristics for which data were extracted (e.g., study size, PICOS, follow-up period) and provide the citations. | Table 1 |
| Risk of bias within studies | 19 | Present data on risk of bias of each study and, if available, any outcome level assessment (see item 12). | 6 & suppl material |
| Results of individual studies | 20 | For all outcomes considered (benefits or harms), present, for each study: (a) simple summary data for each intervention group (b) effect estimates and confidence intervals, ideally with a forest plot. | Figure 2.1-2.2 |
| Synthesis of results | 21 | Present results of each meta-analysis done, including confidence intervals and measures of consistency. | 11-12 |
| Risk of bias across studies | 22 | Present results of any assessment of risk of bias across studies (see Item 15). | N.A. |
| Additional analysis | 23 | Give results of additional analyses, if done (e.g., sensitivity or subgroup analyses, meta-regression [see Item 16]). | N.A. |
| **DISCUSSION** | | |  |
| Summary of evidence | 24 | Summarize the main findings including the strength of evidence for each main outcome; consider their relevance to key groups (e.g., healthcare providers, users, and policy makers). | 13 |
| Limitations | 25 | Discuss limitations at study and outcome level (e.g., risk of bias), and at review-level (e.g., incomplete retrieval of identified research, reporting bias). | 14-15 |
| Conclusions | 26 | Provide a general interpretation of the results in the context of other evidence, and implications for future research. | 15 |
| **FUNDING** | | |  |
| Funding | 27 | Describe sources of funding for the systematic review and other support (e.g., supply of data); role of funders for the systematic review. | 16 |

*From:*  Moher D, Liberati A, Tetzlaff J, Altman DG, The PRISMA Group (2009). Preferred Reporting Items for Systematic Reviews and Meta-Analyses: The PRISMA Statement. PLoS Med 6(7): e1000097. doi:10.1371/journal.pmed1000097

**Methods**

**Oral antibiotics for neonatal infections: a systematic review and meta-analysis**

**Search strategy**

*Research question:* Is intravenous to oral switch antibiotic therapy as safe and effective as intravenous therapy in clinically stable neonates or children with a bacterial infection?

*Used databases:* Embase.com; Medline Epub (Ovid), Cochrane Central, Web of Science, Google Scholar

**Results**

| *Database* | *Number of refs* | *Refs after deduplication* |
| --- | --- | --- |
|  |  |  |
| Embase.com | 2439 | 2409 |
| Medline Epub (Ovid) | 1353 | 532 |
| Cochrane Central | 107 | 37 |
| Web of Science | 460 | 129 |
| Google Scholar | 200 | 131 |
|  |  |  |
| ***Total*** | ***4559*** | ***3238*** |

*Deduplicated: 1321*

*Update Jan 22^nd^ 2019 – Feb 6^th^ 2018: 170*

***Embase.com***

**(**'child'/exp OR pediatrics/exp OR childhood/exp OR 'child development'/de OR 'child growth'/de OR 'child health'/de OR 'child health care'/exp OR 'child care'/exp OR 'perinatal care'/de OR 'childhood disease'/exp OR 'child death'/exp OR 'pediatric ward'/de OR 'pediatric hospital'/de OR 'pediatric nursing'/exp OR 'pediatric anesthesia'/de OR 'pediatric intensive care unit'/de OR 'neonatal intensive care unit'/de OR 'newborn sepsis'/de OR 'perinatal infection'/de OR (infan* OR newborn* OR perinatal* OR (new NEXT/1 born*) OR baby OR babies OR neonat* OR child* OR kid OR kids OR toddler* OR teen* OR boy* OR girl* OR minors OR underag* OR (under NEXT/1 (age* OR aging)) OR pediatric* OR paediatric* OR school* OR preschool*):ab,ti**) AND (**'antibiotic therapy'/de OR 'antibiotic agent'/exp OR (antibiotic* OR antibacter* OR ((anti) NEXT/1 (biotic* OR bacter*)) OR aminoglycosid* OR gentamicin* OR tobramycin* OR penicillin* OR amoxicillin* *OR ampicillin**):ab,ti**) AND (**'enteral drug administration'/exp OR (oral OR oropharyngeal OR buccal OR ((enteral*) NEAR/3 (administrat* OR therap* OR treatment* OR medication* OR deliver* OR antibiotic* OR amoxicillin* OR dose* OR dosage*)) OR 'per os'):ab,ti**) AND (**'intravenous drug administration'/de OR 'parenteral drug administration'/de OR 'intramuscular drug administration'/de OR (intravenous* OR intramuscular* OR infusion* OR vein* OR venous* OR ((parenteral* OR IV) *NEAR/6* (administrat* OR therap* OR treatment* OR medication* OR infusion* OR deliver* OR antibiotic* OR amoxicillin* OR dose* OR dosage*)) OR inject* OR switch):ab,ti**) AND (**'sepsis'/exp OR 'bacterial infection'/exp OR 'perinatal infection'/de OR (sepsis OR septic* OR bacteremi* OR bacillemi* OR bacillaem* OR bacteria* OR bacilla* OR ((Gram) NEXT/1 (positive OR negative)) OR ((perinatal OR neonat* OR newborn*) NEXT/1 (infecti*)) OR 'clinically stable'):ab,ti**) NOT (**[animals]/lim NOT [humans]/lim**) NOT (**'Conference abstract'/it OR 'Editorial'/it**)**

***Medline (Ovid)***

(exp "Infant, Newborn"/ OR exp "Child"/ OR exp "Pediatrics"/ OR exp "Child Development"/ OR exp "Child Health"/ OR exp "Child Care"/ OR "Hospitals, Pediatric"/ OR exp "Pediatric Nursing"/ OR exp "Intensive Care Units, Pediatric"/ OR "Neonatal Sepsis"/ OR "Perinatal Death"/ OR exp "Infant Death"/ OR (infan* OR newborn* OR (new ADJ1 born*) OR baby OR babies OR neonat* OR child* OR kid OR kids OR toddler* OR teen* OR boy* OR girl* OR minors OR underag* OR (under ADJ (age* OR aging)) OR pediatric* OR paediatric* OR school* OR preschool*).ab,ti.) **AND** (exp "Anti-Bacterial Agents"/ OR (antibiotic* OR antibacter* OR ((anti) ADJ1 (biotic* OR bacter*)) OR aminoglycosid* OR gentamicin* OR tobramycin* OR penicillin* OR amoxicillin* *OR ampicillin**).ab,ti.) **AND** (exp "Administration, Oral"/ OR (oral* OR oropharyngeal* OR buccal* OR ((enteral*) ADJ3 (administrat* OR therap* OR treatment* OR medication* OR deliver* OR antibiotic* OR amoxicillin* OR dose* OR dosage*)) OR "per os").ab,ti.) **AND** (exp "Administration, Intravenous"/ OR "Infusions, Parenteral"/ *OR 'intramuscular drug administration'/de* OR (intravenous* *OR intramuscular** OR infusion* OR vein* OR venous* OR ((parenteral* OR IV) *ADJ6* (administrat* OR therap* OR treatment* OR medication* OR infusion* OR deliver* OR inject* OR antibiotic* OR amoxicillin* OR dose* OR dosage*)) *OR inject* OR switch*).ab,ti.**) AND (**exp "Sepsis"/ OR exp "Bacterial Infections"/ OR (sepsis OR septic* OR bacteremi* OR bacillemi* OR bacillaem* OR bacteria* OR bacilla* OR ((Gram) ADJ1 (positive OR negative)) OR ((perinatal OR neonat* OR newborn*) ADJ1 (infecti*)) *OR "clinically stable"*).ab,ti.**)** **NOT** (exp animals/ NOT humans/) **NOT (**congresses.pt. OR editorial.pt.**)**

***Cochrane Central (trials)***

**(**(infan* OR newborn* OR perinatal* OR (new NEXT/1 born*) OR baby OR babies OR neonat* OR child* OR kid OR kids OR toddler* OR teen* OR boy* OR girl* OR minors OR underag* OR (under NEXT/1 (age* OR aging)) OR pediatric* OR paediatric* OR school* OR preschool*):ab,ti**) AND (**(antibiotic* OR antibacter* OR ((anti) NEXT/1 (biotic* OR bacter*)) OR aminoglycosid* OR gentamicin* OR tobramycin* OR penicillin* OR amoxicillin* *OR ampicillin**):ab,ti**) AND (**(oral OR oropharyngeal OR buccal OR ((enteral*) NEAR/3 (administrat* OR therap* OR treatment* OR medication* OR deliver* OR antibiotic* OR amoxicillin* OR dose* OR dosage*)) OR 'per os'):ab,ti**) AND (**(intravenous* *OR intramuscular** OR infusion* OR vein* OR venous* OR ((parenteral* OR IV) *NEAR/6* (administrat* OR therap* OR treatment* OR medication* OR infusion* OR deliver* OR inject* OR antibiotic* OR amoxicillin* OR dose* OR dosage*)) *OR inject* OR switch*):ab,ti**) AND (**(sepsis OR septic* OR bacteremi* OR bacillemi* OR bacillaem* OR bacteria* OR bacilla* OR ((Gram) NEXT/1 (positive OR negative)) OR ((perinatal OR neonat* OR newborn*) NEXT/1 (infecti*)) *OR 'clinically stable'*):ab,ti**)**

***Web of Science***

**TS=((**(infan* OR newborn* OR perinatal* OR (new NEAR/1 born*) OR baby OR babies OR neonat* OR child* OR kid OR kids OR toddler* OR teen* OR boy* OR girl* OR minors OR underag* OR (under NEAR/1 (age* OR aging)) OR pediatric* OR paediatric* OR school* OR preschool*)**) AND (**(antibiotic* OR antibacter* OR ((anti) NEAR/1 (biotic* OR bacter*)) OR aminoglycosid* OR gentamicin* OR tobramycin* OR penicillin* OR amoxicillin* *OR ampicillin**)**) AND (**(oral OR oropharyngeal OR buccal OR ((enteral*) NEAR/2 (administrat* OR therap* OR treatment* OR medication* OR deliver* OR antibiotic* OR amoxicillin* OR dose* OR dosage*)) OR "per os")**) AND (**(intravenous* *OR intramuscular** OR infusion* OR vein* OR venous* OR ((parenteral* OR IV) *NEAR/5* (administrat* OR therap* OR treatment* OR medication* OR infusion* OR deliver* OR inject* OR antibiotic* OR amoxicillin* OR dose* OR dosage*)) *OR inject* OR switch*)**) AND (**(sepsis OR septic* OR bacteremi* OR bacillemi* OR bacillaem* OR bacteria* OR bacilla* OR ((Gram) NEAR/1 (positive OR negative)) OR ((perinatal OR neonat* OR newborn*) NEAR/1 (infecti*)) *OR "clinically stable"*)**)**) **AND** DT=Article

***Google Scholar: 200*** *(top relevant references)*

infant|newborn|neonate|neonatal|child|children|pediatric|paediatric antibiotic|antibacterial|aminoglycoside|gentamicin|tobramycin|penicillin|amoxicillin oral|enteral intravenous|intramuscular|infusion|venous|*injectable* sepsis|septic|bacteremia|bacterial

**Oral antibiotics for neonatal infections: a systematic review and meta-analysis: Data extraction form**

Name of first author, year of publication:

Country of origin:

1. Study characteristics

- - Study design
  - Study size
  - Patient characteristics: gestational age, postnatal age
  - Clinical indication for antibiotic therapy: yes/no, If yes what condition/definition of sepsis
  - Aim of study

2. Intervention

- Type of antibiotic regimen
- Route of administration
- Single dose/multiple dosing
- Duration of therapy

3. Comparison

- Was there a comparison/control group? If yes:
  - Patient characteristics: gestational age, postnatal age
  - Type of antibiotic regimen
  - Route of administration
  - Single dose/multiple dosing
  - Duration of therapy

4. Primary outcome

- If: Mortality/treatment failure 🡪 extract data

5. Secondary outcomes + results

6. Pharmacokinetic analysis: yes/no

- - Dosage regimen
  - Sampling frequency
  - Measured parameters

**Table S1: overview of used Quality assessment tools per included study**

| **Included studies** | **Used Quality assessment Scale** |
| --- | --- |
| Autret et al (1988 & 1989) | Cochrane Risk of Bias Tool/ClinPK tool |
| Bang et al (1999 & 2005) | NOS Scale |
| Baqui et al (2015) | Cochrane Risk of Bias Tool |
| Blond et al (1990) | NOS Scale |
| Coffey et al (2012) | NOS Scale |
| Cohen et al (1975) | ClinPK tool |
| Degefie Hailegebriel et al (2017) | Cochrane Risk of Bias Tool |
| Giustardi et al (1992) | Cochrane Risk of Bias Tool |
| Gras le Guen et al (2007) | ClinPK tool |
| Grossman et al (1966) | ClinPK tool |
| Herngren et al (1987) | ClinPK tool |
| Huang et al (1953) | ClinPK tool |
| Lönnerholm (1982) | ClinPK tool |
| Magín et al (2007) | *Retrospective study* |
| Manzoni et al (2009) | NOS Scale |
| Mir et al (2013) | ClinPK tool |
| Mir et al (2017) | Cochrane Risk of Bias Tool |
| Mulhall et al (1985) | ClinPK tool |
| O’Connor et al (1965) | ClinPK tool |
| Qamar et al (2013) | NOS Scale |
| Squinazi et al (1983) | ClinPK tool |
| Sicard et al (2015) | ClinPK tool |
| Silverio et al (1973) | ClinPK tool |
| Tikmani et al (2017) | Cochrane Risk of Bias Tool |
| Tshefu et al (2015) | Cochrane Risk of Bias Tool |
| Tshefu et al (2015) | Cochrane Risk of Bias Tool |
| Weber et al (1999) | ClinPK tool |
| Weingärtner et al (1977) | ClinPK tool |
| Zaidi et al (2012) | Cochrane Risk of Bias Tool |

**Table S2 ClinPK Checklist & Quality assessment**

|  | **Checklist Item** |
| --- | --- |
|  | **Title/Abstract** |
| 1. | The title identifies the drug(s) and patient population(s) studied. |
| 2. | The abstract minimally includes the name of the drug(s) studied, the route of administration, the population in whom it was studied, and the results of the primary objective and major clinical pharmacokinetic findings. |
|  | **Background** |
| 3. | Pharmacokinetic data (i.e., absorption, distribution, metabolism, excretion) that [are] known and relevant to the drugs being studied [are] described. |
| 4. | An explanation of the study rationale is provided. |
| 5. | Specific objectives or hypotheses [are] provided. |
|  | **Methods** |
| 6 | Eligibility criteria of study participants are described. |
| 7. | Co-administration (or lack thereof) of study drug(s) with other potentially interacting drugs or food within this study is described. |
| 8. | Drug preparation and administration characteristics including dose, route, formulation, infusion duration (if applicable), and frequency are described. |
| 9. | Body fluid or tissue sampling (timing, frequency, and storage) for quantitative drug measurement is described. |
| 10. | Validation of quantitative bioanalytical methods used in the study [is] referenced or described if applicable. |
| 11. | Pharmacokinetic modeling methods and software used are described, including assumptions made regarding the number of compartments and order of kinetics (zero, first, or mixed order). |
| 12. | For population pharmacokinetic studies, covariates incorporated into pharmacokinetic models are identified and described. |
| 13. | Formulas for calculated variables (such as creatinine clearance, body surface area, AUC, and adjusted body weight) are provided or referenced. |
| 14. | The specific body weight used in drug dosing and pharmacokinetic calculations [is] reported (i.e., ideal body weight versus actual body weight versus adjusted body weight). |
| 15. | Statistical methods including software used are described. |
|  | **Results** |
| 16. | Study withdrawals or subjects lost to follow-up (or lack thereof) are reported. |
| 17. | Quantification of missing or excluded data is provided if applicable. |
| 18. | All relevant variables that may explain inter- and intra-patient pharmacokinetic variability (including: age, sex, end-organ function, ethnicity, weight or BMI, health status or severity of illness, and pertinent co-morbidities) are provided with appropriate measures of variance. |
| 19. | Results of pharmacokinetic analyses are reported with appropriate measures of precision (such as range or 95% confidence intervals). |
| 20. | Studies in patients receiving extracorporeal drug removal (i.e., dialysis) should report the mode of drug removal, type of filters used, duration of therapy, and relevant flow rates. |
| 21. | In studies of drug bioavailability comparing two formulations of the same drug, F (bioavailability), AUC, Cmax (maximal concentration), and Tmax (time to maximal concentration) should be reported. |
|  | **Discussion/Conclusion** |
| 22. | Study limitations describing potential sources of bias and imprecision where relevant should be described. |
| 23. | The relevance of study findings (applicability, external validity) is described. |
|  | **Other Information** |
| 24. | Funding sources and conflicts of interest for the authors are disclosed. |

**Table S3. ClinPK and checklist results**

|  | **Autret et al, 1988** | **Autret et al, 1989** | **Cohen et al, 1975** | **Giustardi et al, 1992** | **Gras le Guen et al, 2007** | **Grossman et al, 1965** | **Herngren et al, 1987** | **Huang et al, 1953** | **Lönnerholm et al, 1982** |
| --- | --- | --- | --- | --- | --- | --- | --- | --- | --- |
| **1.** | Yes, amoxicillin | Yes, amoxicillin | Yes | Yes, amoxicillin | Yes, amoxicillin | Yes, all mentioned | Yes, flucloxacillin | Yes, penicillin | Yes |
| **2.** | Yes, complete abstract provided | No, letter to the editor, no absract | Yes, complete abstract provided | Yes, complete abstract provided | Yes, complete abstract provided | Yes, abstract complete provided | Yes, abstract provided | No abstract provided | Yes, complete abstract provided |
| **3.** | Yes | No, only refers to previos performed study | Yes | No references used in introduction | Yes, in introduction | Yes | Yes, some background in introduction | Yes, short introduction | Yes |
| **4.** | Yes, rationale is clear | Yes, rationale is clear | Yes | Yes, rationale is clear | Yes, rationale is clear | Yes, rationale is clear | Yes, rationale is clear | Yes, rationale is clear | Yes, rationale is clear |
| **5.** | Yes, objective is mentioned | Yes, objective is mentioned | Yes/ok | Yes | Yes, objective is mentioned | Yes, objectives | Yes | Yes | Yes |
| **6.** | Yes | Yes, inclusion is described | Yes | Yes, inclusion is described | Yes, inclusion & exclusion clearly described | Yes | Yes | No detailed inclusion criteria | Yes |
| **7.** | Yes, initial therapy described | Initial therapy mentioned, no co-medication mentioned | No | No | Yes, initial therapy is described | ± not more than 1 single dose of studied ab | Yes, mentioned that no other drugs were administrated | Not mentioned | Yes, switch study, no co-medication mentioned |
| **8.** | Yes | Yes | Yes | Yes | Yes, section dosage | Yes | Yes | Yes | Yes |
| **9.** | Yes | Yes | Yes | Yes | Yes | Yes | Yes | Yes | Yes |
| **10.** | HPLC, Pharm program Gomeni | HPLC/MS method | Agar plate method  Sarcina Lutea | HPLC | HPLC, LiChorspher | Agar diffusion method | Disc diffusion method S. Aureus  Equilibrium dialysis | Dilution method. Agar plate method | Agar plate method  Staf epidermidis |
| **11.** | N.A. | No | Mathematical method of Bennett et al 1966 | No | No | No | 2 compartment equation, one compartment open body kinetic analysis | No | No |
| **12.** | N.A. | N.A. | N.A. | N.A. | N.A. | N.A. | Covariates? | N.A. | N.A. |
| **13.** | N.A. | N.A. | No | N.A. | Not mentioned | description of serum levels | Yes, formulas provided | No | Yes |
| **14.** | N.A. | N.A. | N.A. | N.A. | N.A. | N.A. | N.A. | N.A. | N.A. |
| **15.** | Student T test, software not specified | No, no software mentioned | Not provided | No | Yes, statisical section | Not provided | Yes, student’s T-test | Not provided | Not provided |
| **16.** | N.A. | N.A. | N.A. | N.A. | Not described | N.A. | N.A. | N.A. | N.A. |
| **17.** | N.A. | N.A. | N.A. | N.A. | N.A. | N.A. | N.A. | N.A. | N.A. |
| **18.** | Yes, table 1 | Yes | Yes, age, gestation and birth weight provided | Yes | Yes | No, described in inclusion criteria | Yes, provided in table 1 | No information provided except age | No, no information provided |
| **19.** | Mean + SD | Mean + SD + range | Mean + SD + range | Median + SD | Mean + SD | Mean + range | Mean + SD | Mean/median + range | Mean + range |
| **20.** | N.A. | N.A. | N.A. | N.A. | N.A. | N.A. | N.A. | N.A. | N.A. |
| **21.** | T ½, distribution volume, AUC, Cmax | No | No | No | No | No | All reported | No | AUC, bioavailability reported |
| **22.** | No | No | Yes | No | Yes | ± | Yes | Yes | Yes |
| **23.** | Yes | No | Yes | Yes | Yes | Yes | Yes | No | Yes |
| **24.** | No | No | No | No | No | Yes | Yes | No | No |

**Table S3 continued**

|  | **Mir et al, 2013** | **Mulhall et al, 1985** | **O’ Connor et al, 1965** | **Sicard et al, 2015** | **Silverio et al, 1973** | **Squinazi et al, 1983** | **Weber et al,**  **1999** | **Weingartner et al, 1977** |
| --- | --- | --- | --- | --- | --- | --- | --- | --- |
| **1.** | Yes, amoxicillin | Antibiotic type not further specified | Yes, nafcillin | Yes, linezolid | Yes | Yes, amoxicillin | Yes, chloramphenicol | Yes, amoxicillin |
| **2.** | Yes, complete abstract provided | Yes, complete abstract provided | Yes, brief abstract provided | Yes, complete abstract provided | No abstract provided | Yes, complete abstract provided | Yes, complete abstract provided | Yes, complete abstract provided |
| **3.** | Yes, in introduction | Yes | Yes, one reference mentioned in introduction | Yes, background in introduction | Yes, review of previous work | Yes, in introduction | Yes, in introduction | Yes, in introduction |
| **4.** | Yes, rationale is clear | Yes | Yes, rationale is clear | Yes | Partly, mentioned later in the paper | Yes, rationale is clear | Yes, rationale is clear | Yes, rationale is clear |
| **5.** | Yes | Yes | Yes | Yes | partly | Yes, 3 objectives are mentioned | Yes | Yes |
| **6.** | Yes, inclusion is described | Yes | No inclusion criteria | Yes, inclusion is described | Yes | Yes | Yes, inclusion is described | Ok, not extensively described |
| **7.** | No, co-medication not described, there was no initial therapy | Yes, first line therapy at start study | no | Yes, previous treatment provided | No, no co-medication or initial therapy mentioned | Yes, initial therapy is described | Previous treatment with antibiotics was exclusion | No, co-medication not specified (healthy newborn) |
| **8.** | Yes | Yes | Yes | Yes | Yes | Yes, conduite du traitement | Yes | Yes |
| **9.** | Yes | Yes | Yes | Yes | Yes | Yes | Yes | No, not clearly described |
| **10.** | HPLC/MS method | Microbiological plate diffusion techniques | Agar cup diffusion method Sarcina Lutea | HPLC | Agar diffusion method | Microbiological plate diffusion techniques | HPLC | Agar-gel diffusion process |
| **11.** | Agilent Chemstation 32 bit V B.03.01 software | Fortran IV computer program CSTRIP | No | Empower 2 | No | No | No | No |
| **12.** | N.A. | One & two compartment model, no covariates described | N.A. | N.A. | N.A. | N.A. | N.A. | N.A. |
| **13.** | Yes | Yes | N.A. | No | N.A. | N.A. | N.A. | N.A |
| **14.** | N.A. | N.A. | N.A. | N.A. | N.A. | N.A. | N.A. | N.A. |
| **15.** | No, no software mentioned | Yes, student’s t test | No | Stat Soft Inc.  Mann Whitney/chi-squared & Fisher’s exact | Not provided | No | Not provided | No |
| **16.** | N.A. | N.A. | N.A. | N.A. | N.A. | N.A. | N.A. | N.A. |
| **17.** | N.A. | N.A. | N.A. | N.A. | N.A. | N.A. | N.A. | N.A. |
| **18.** | Yes | Yes not all, post-natal age, gestational age | ± | Yes, provided in table 1 | No, no information provided | No | ± |  |
| **19.** | Yes, Mean + SD | Mean + SE | Mean | Mean + SD | mean | Mean + SD + range | % of childen above range | Mean + SD |
| **20.** | N.A. | N.A. | N.A. | N.A. | N.A. | N.A. | N.A. | N.A. |
| **21.** | Yes | Yes | No | No, retrospective report | No | No | Yes | T ½ |
| **22.** | Yes | Yes | Yes | Yes | Yes | Ok | Yes | No |
| **23.** | Yes | Yes | Yes | Yes | Yes | yes | Yes | Yes |
| **24.** | No | Ok, no disclosure | Acknowledgment | Yes | Acknowledgement | No | Yes | No |

**Table S4 Quality assessment of Clinical studies using the NewCastle-Ottowa Quality Assessment Scale (NOS)**

| **RISK OF BIAS ASSEMENT: NEWCASTLE-OTTAWA QUALITY ASSESSMENT SCALE** | | | | | | | | | | | | |
| --- | --- | --- | --- | --- | --- | --- | --- | --- | --- | --- | --- | --- |
| **Selection** | | | | | | **Comparability** | | **Outcome** | | | |  |
| *Study ID, year* | *Represen-tativeness of the exposed cohort* | *Selection of the non-exposed cohort* | *Ascertain-ment of exposure* | *Outcome not present at start* | *Total* | *Comparability of cohorts on basis of design or analysis* | *Total* | *Asses-ment of outcome* | *Follow-up long enough for outcome* | *Ade-quacy of follow up* | *Total* | *Quality** |
| Bang et al, 1999 | * | * | * | * | 4 |  | 0 | * | * | * | 3 | Good |
| Blond et al, 1990 | * |  | * | * | 3 |  | 0 |  | * | * | 2 | Fair |
| Coffey et al, 2012 | * |  | * | * | 3 |  | 0 |  |  |  | 0 | poor |
| Manzoni et al, 2009 | * | * | * | * | 4 | * | 1 |  | * | * | 2 | Good |
| Qamar et al, 2013 | * |  | * | * | 3 |  | 0 |  | * | * | 3 | Fair |

**Table S5. Comparison of studies**

| **Selection** | | | | | **Comparability** | **Outcome** | | |
| --- | --- | --- | --- | --- | --- | --- | --- | --- |
| *Study ID, year* | *Representativeness of the exposed cohort* | *Selection of the non-exposed cohort* | *Ascertainment of exposure* | *Outcome not present at start* | *Comparability of cohorts on basis of design or analysis* | *Assessment of outcome* | *Follow-up long enough for outcome* | *Adequacy of follow up* |
| Bang et al  1999 | A: 39 villages from Gadchiroli district in India | A: 47 control villages drawn from same district | A: trained health workers who were trained and equipped with an neonatal kit visited the intervention villages | A: primary outcome is mortality | Villages from the same district with similar socio-demographic characteristics and mortality rates. No random selection of villages. | A: mortality was assessed by an independent neonatologist | A: Follow up of 28 days is long enough to assess neonatal mortality | A: Follow up consisted of home visits |
| Blond et al  1990 | A: newborn with suspected bacterial infection | C: No non-exposed cohort included | A: antibiotic treatment in hospital | B: did not receive oral AB therapy before start study | N.A. | B: questionnaires to be filled in by parents after 1 month. | A: follow up of 1 month is adequate for assessment. | B: 87.4 % of questionnaires were returned. |
| Coffey et al 2012 | A:newborn < 59 days, suspected of possible severe bacterial infection | C: No non-exposed cohort included | A: trained health workers + supervisor administered medication | A: did not receive treatment before enrolment | N.A. | D: no description. | B: not specified. | D: no statement. |
| Manzoni et al  2009 | A: full term newborn with presumed or proven infection | A: matched controls, same clinical condition | A: switch from IV to oral therapy after 72 hours of IV therapy, controls continued with IV therapy | A: primary outcomes: clinical deterioration, reduction of stay, breast feeding, IV cannulas | A: matched for gender, gestational age, birth weight, type of feeding, age at diagnosis | D: no description provided | A: short follow up but adequate for predefined outcome measurements | A: no loss to follow up |
| Qamar et al  2013 | A: cases of omphalitis in first 2 months of live | C: no non-exposed cohort included | A: instruction for administration was given by the health worker to mother/caregiver. | A: primary outcomes was decreased redness/cellulitis or decreased discharge, resolution of symptoms or no improvement after 48 hours | N.A. | C: outcome was assessed by physicians at the clinic, not further described. | A: yes, 7 days | A: loss to follow up of 0.18%. |

**Figure S1. Quality Assessment of RCT’s using the Cochrane Risk of Tool**


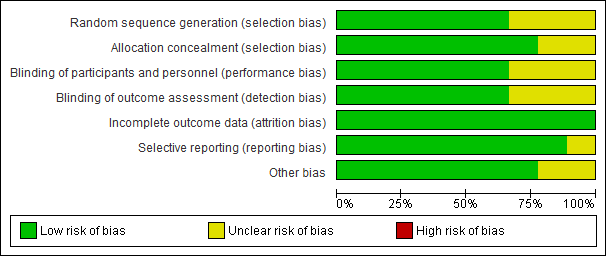

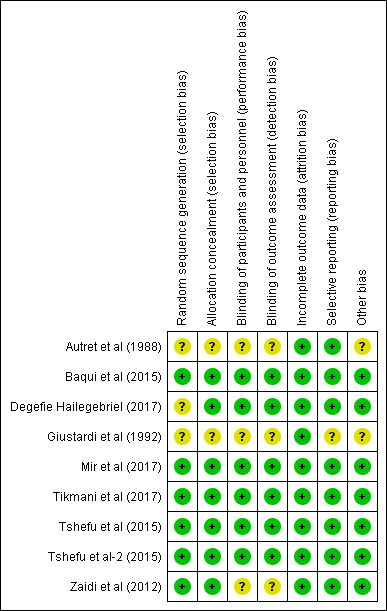

Supplement: dkz252_Supplementary_Data [file dkz252_supplementary_data.docx]
